# The next generation of obesity treatments: beyond suppressing appetite

**DOI:** 10.3389/fpsyg.2013.00721

**Published:** 2013-10-09

**Authors:** Nicole M. Avena, Susan Murray, Mark S. Gold

**Affiliations:** ^1^Department of Psychiatry, University of FloridaGainesville, FL, USA; ^2^Department of Psychology, Princeton UniversityPrinceton, NJ, USA

**Keywords:** obesity, appetite suppressants, food addiction, palatable food, animal models

Obesity remains a prominent public health concern in the United States as well as many other countries, with 33% of adults worldwide overweight or obese in 2005 and an estimated 60% by 2030 (Kelly et al., 2008). This data highlights the need for effective prevention and intervention strategies. Obesity can be viewed as an endpoint with many possible contributing factors, including genetic propensity, sedentary lifestyles, and the relative ease with which one can obtain food, particularly in modern industrialized societies. Such factors may result in an imbalance between the number of calories consumed vs. expended. The majority of pharmaceutical compounds that have been developed to combat obesity aim to correct or improve this last factor by suppressing appetite (See Table 1). However, the number of people with obesity in the United States does not appear to be decreasing (Flegal et al., 2012). Here we present some possible reasons why these drugs have failed to fully address the problem of obesity.

**Table 1:** **Current pharmaceutical treatments of obesity**.

**Name**	**Approved**	**Type of compound**	**Proposed mechanism of action**
Phentermine	1959	Sympathomimetic amine	Appetite suppressant
Fenfluramine^*^, Dexfenfluramine^*^		Serotonergic agent	Appetite suppressant
Benzphetamine		Sympathomimetic amine	Appetite suppressant
Phendimetrazine	1979	Sympathomimetic amine	Appetite suppressant
Diethylpropion		Sympathomimetic amine	Appetite suppressant
Sibutramine^*^	1997	Noradrenaline and serotonin reuptake inhibitor	Appetite suppressant (also stimulates thermogenesis)
Orlistat	1999	Lipase inhibitor	Inhibits the absorption of dietary fat
Phentermine/Topiramate	2012	Sympathomimetic amine/sulfamate-substituted monosaccharide	Appetite suppressant
Lorcaserin	2012	Selective serotonin 2C receptor agonist	Appetite suppressant

* Removed from the market.

First, the weight loss associated with the appetite suppressant drugs on the market is not as dramatic as one might expect. For example, one drug which received FDA approval in 2012, Belviq, has been found to result in a mean weight loss of 3–3.6%, corrected for the effects of placebo, in phase III clinical trials (Miller, 2013). Another recently approved drug, Qsymia, which consists of a combination of phentermine and extended-release topiramate, an anti-epileptic drug, has shown greater effects, producing a mean weight loss of between 7.5 and 9.3%, corrected for the effects of placebo, during phase III clinical trials (Garvey, 2013). While these medications may be more or less effective for certain individuals, especially in light of other lifestyle changes, and these reductions are certainly beneficial, they do not appear to be the definitive weight loss interventions.

Second, suppressing appetite may be too simplistic of an approach. Instead of aiming to reduce intake of all types of foods, it may be more beneficial over the long term to specifically reduce excessive intake of foods rich in fats or sugars, which can contain excessive amounts of calories but offer minimal nutritional value. To our knowledge, no appetite suppressant on the market to date has been known to reduce intake of specific types of food. This approach may facilitate the normalization of eating patterns by decreasing overconsumption of less healthy options without affecting intake of sources of greater nutrition. Further, recent research suggests that the age old adage “a calorie is a calorie” is not exactly true. Not all calories are created equal. For example, research has found that there are differences in the effects of equicaloric diets comprised of different proportions of macronutrients (Ebbeling et al., 2012). Resting and total energy expenditure has been shown, for instance, to be most decreased in response to a low-fat diet, compared to low glycemic index and low carbohydrate diets, without differences in physical activity, suggesting that diets consisting of varying macronutrient compositions can differentially affect one's metabolism. Further, this finding supports the hypothesis that targeted pharmacotherapy may be beneficial.

It is also possible that appetite suppressants may not have worked well for obesity because these medications have targeted appetite in general, rather than targeting the mechanisms of reinforcement associated with food intake, as has been initiated in the past with the endocannabinoid system (Carai et al., 2006). Targeting neural reward systems may also, in turn, result in a selective suppression of appetite for certain types of highly reinforcing foods. Findings from our laboratory and others have revealed that overconsumption, or binge consumption, of sugars and fats can lead to brain and behavioral changes similar to those seen within the context of drug addiction, providing empirical support for the concept of food addiction (Avena et al., 2008; Oswald et al., 2011; Iemolo et al., 2012; Rossetti et al., 2013). Such findings point to one potential reason that weight loss may be so challenging for some, but also provide an avenue to study potential treatments that consider and address the effects that these ingredients can have on the brain. In light of the similarities between binge intake of highly palatable foods and drug addiction, our laboratory and others have been interested in studying the effects of medications that have been used to treat substance use disorders on overeating. For example, the GABA-B agonist, baclofen, which has been found to be effective in treating several aspects of alcohol dependence in many (Addolorato et al., 2000, 2007, 2011) but not all studies (Garbutt et al., 2010), has also been shown to aid in the reduction of binge eating behavior in both preclinical and clinical studies (Buda-Levin et al., 2005; Broft et al., 2007; Berner et al., 2009; Corwin et al., 2012). Additionally, baclofen has shown some success in inhibiting weight gain in animals and promoting weight loss in clinical samples (Sato et al., 2007; Arima and Oiso, 2010; Patel and Ebenezer, 2010).

Using the food addiction model in the laboratory may be an innovative approach to identifying potential weight loss treatments which take into account the neurochemical effects of regularly overeating certain macronutrients. In clinical samples, 25% of participants who are obese meet the diagnostic criteria for “food addiction,” according to the Yale Food Addiction Scale (YFAS) (Davis et al., 2011). Studies assessing obese individuals with binge eating disorder have found that 42–57% participants meet this criteria (Gearhardt et al., 2012, 2013), and the percentage of obese individuals seeking bariatric surgery who meet this criteria is 42% (Meule et al., 2012). Thus, while not all individuals who are obese express addiction-like characteristics or behaviors regarding food, there are a significant number of individuals who do, and who may thus benefit from addiction-targeting treatments. One avenue for this research includes the study of medications that have been successful in treating substance use disorders (Gold and Avena, 2013). With redefined goals in mind, including a greater focus on the reduction of overeating specific foods, such research may lead to the development of more targeted weight loss treatments.
